# Simulated Digestion and Fermentation In Vitro by Obese Human Gut Microbiota of Sulforaphane from Broccoli Seeds

**DOI:** 10.3390/foods11244016

**Published:** 2022-12-12

**Authors:** Yifei Sun, Zhaocheng Tang, Tingting Hao, Zeyu Qiu, Baolong Zhang

**Affiliations:** 1 School of Food and Biological Engineering, Jiangsu University, Zhenjiang 212013, China; 2 Provincial Key Laboratory of Agrobiology and Institute of Germplasm Resources and Biotechnology, Jiangsu Academy of Agricultural Sciences, Nanjing 210014, China

**Keywords:** sulforaphane, digestion, fermentation, gut microbiota, short-chain fatty acids

## Abstract

Background: sulforaphane is a kind of isothiocyanate, which is obtained by hydrolysis of glucosinolate by the unique myrosinase in plants. It has been proved to prevent the occurrence of many chronic diseases, such as obesity, diabetes and cancer. Objective: The impact of SFN on obese human gut flora, however, has not been established. Methods: In this research, SFN was isolated from broccoli seeds and then refined to achieve 95% purity. Next, an investigation was conducted into the digestion and fermentation processes of SFN. Results: The stability of the SFN in simulated saliva, gastric fluid, and intestinal juice provides evidence that it can reach the gut and be available for utilization by gut microflora. In vitro fermentation of SFN by gut microbes in obese patients results in alteration in constitution of microbiota and production of short chain fatty acids. As the result of SFN ingestion by human gut bacteria, the content of butyric and valeric acids increased 1.21- and 1.46-fold, respectively. In obese human guts, the relative abundances of the beneficial genera including *Lactobacillus*, *Weissella*, *Leuconosto*, *Algiphilus* and *Faecalibacterium* significantly increased, whilst the detrimental genera, such as *Escherichia-Shigella*, *Klebsiella*, *Clostridium_sensu_stricto_1*, *Sutterella*, *Megamonas* and *Proteus* drastically declined. Conclusion: Taken together, these findings demonstrate that SFN can be used as a nutraceutical ingredient for obese patients and for improving human health.

## 1. Introduction

The human gut provides a good living environment for microorganisms, so that the human body has metabolic functions that it does not have. The gut is the largest immune organ in the human body and plays an extremely important role in maintaining normal immune defense functions. The gut microbiota is a diverse microbial community in the intestinal tract, which encompasses a myriad variety of bacteria and a small number of archaea. At the phylum level, the *Bacteroidetes*, *Firmicutes*, *Actinobacteria* and *Proteobacteria* represent the primary bacteria in most mammals [1]. Intestinal flora can be divided into two categories: probiotics and harmful bacteria. Probiotics include *Bifidobacterium*, *Lactobacillus*, *Bacillus licheniformis*, etc., which can synthesize multivitamins needed by the human body, such as B vitamins, vitamin K, etc., which are helpful for food absorption and digestion, and can promote body metabolism and enhance immunity [2,3]; harmful bacteria include *Clostridium perfringens*, *Staphylococcus aureus*, etc. Decreased immunity and uncontrolled growth can lead to various diseases, such as colitis or psoriasis, and may also affect the normal functions of the human liver, nerves and blood vessels [4].

The intestinal flora obtains energy by fermenting the ingested food, produces vital metabolites, such as the short-chain fatty acids (SCFAs), and fosters the development and maturation of the host immune system. SCFAs are among the most thoroughly investigated bacterial metabolites, *Bacteroides* provides most of the acetate and propionate, and *Firmicutes* is considered the main producer of butyrate [5]. Acetic, propionic, and butyric acids provide energy to host tissues and have anti-inflammatory properties in the intestinal mucosa. SCFAs may affect the regulation of adipogenesis and also play an important role in determining the intestinal environment, being able to influence intestinal transit and microbial balance [6]. The gut microbes themselves and their metabolites can promote digestion and absorption and synthesize a variety of vitamins, but a disrupted microbiome can cause inflammatory bowel disease, cancer, and metabolic disease [7], and may also increase the incidence of obesity and type 2 diabetes [8]. The health of the human body is closely related to the intestinal flora, and maintaining the dynamic balance of intestinal microorganisms plays a very important role in maintaining a healthy state of the body. Therefore, managing a healthy gut environment by preserving the equilibrium of the beneficial microbiota and reducing the populations of harmful bacterial strains may prove to be a useful method in the field of preventive medicine.

Sulforaphane (SFN) is an isothiocyanate, which is abundant in cruciferous plants, such as broccoli, cabbage, cauliflower, and radish. It is produced by glucoraphanin hydrolysis by endogenous myrosinase [9]. A plethora of studies conducted over the past several decades have demonstrated that SFN can be effectively used to treat numerous chronic diseases, such as diabetes, autism spectrum disorder and nonalcoholic fatty liver [10,11]. For obesity, SFN suppressed the expression of transcription factors proliferator-activated receptor γ (PPARγ) and CCAAT/enhancer-binding protein *α* (C/EBP-*α*) and also activated the AMPK pathway in epididymal adipose tissue, thus inhibiting adipose formation [12]. According to relevant literature, SFN has been proved to be an effective bioactive substance extracted from cruciferous plants. SFN can reduce the risk of various malignant tumors, such as breast, bladder, prostate, stomach and colon cancers, by regulating carcinogen metabolic enzymes, blocking the cell cycle, and preventing tumor cell metastasis [13,14,15]. Experiments in vivo show that SFN can reduce the incidence of bladder cancer, diabetes and colitis by restoring the perturbed gut microbiota composition; however, it has not been determined whether the therapeutic effect on obesity is related to the gut microbiota [16,17,18]. In vitro experiments showed that SFN had antibacterial effects against an eclectic range of intestinal pathogenic microorganisms and foodborne pathogens, including *Escherichia coli*, *Listeria monocytogenes* and *Helicobacter pylori* [19,20,21]. However, there are limited reports about the digestion and fermentation of SFN in the colon. In contrast to the disadvantages of high cost, long experiment duration and low reliability associated with the application of an in vivo digestion model, the use of an in vitro digestion model reactor to simulate human intestine has become increasingly popular and is widely employed in studying polysaccharides [22]. The in vitro digestion model encompasses static and dynamic models. The former is the most commonly used in vitro digestion model but is restricted to simple digestion studies, such as starch hydrolysis in rice [23], whereas the latter can be used to analyze more complex digestive systems, particularly the role of fluid dynamics on digestibility. There have been numerous dynamic models with different advantages, such as the TNO’s in vitro large intestinal model, the human gastric simulator (HGS), the gastric digestion simulator, the TNO-advanced gastric model and the bionic gastrointestinal reactor (BGR) [24,25,26,27,28]. Using the BGR, we explored the digestion and fermentation properties of SFN to provide additional evidence for elucidating its disease resistance mechanism.

In this study, we first extracted SFN of high purity from broccoli seeds. The effects of simulated saliva and gastrointestinal juice on SFN was then examined. By fermenting the fecal microbiota of obese patients in the bionic gastrointestinal reactor with medium containing SFN, the effect of SFN on the composition of gut microbiota and production of short-chain fatty acids (SCFAs) was examined. Our study will provide a conceptual underpinning for future work on SFN’s potential application in the nutraceutical industry as a functional ingredient.

## 2. Materials and Methods

### 2.1. Materials and Chemicals

The seeds of broccoli (*Brassica oleracea* var. italica) were purchased from Jiangsu Zhongjiang Seed Industry Co., Ltd. (Nanjing, China). The chemical standard of SFN was purchased from Sichuan Purechem Standard Biotechnology Co., Ltd. (Chengdu, Sichuan, China). Oxoid Brain Heart Infusion (BHI) broth was purchased from Thermo Fisher Scientific (Waltham, MA, USA). Gastric lipase, α-amylase, pepsin, pancreatin, trypsin and bile salt were purchased from Solarbio Science & Technology Co., Ltd. (Beijing, China). SCFAs, including acetic, propionic, n-butyric, i-butyric, n-valeric and i-valeric acids, were purchased from Sinopharm Chemical Reagent Co., Ltd. (Beijing, China). Mucin was purchased from Huhui Biotechnology Co., Ltd. (Shanghai, China). Every other chemical reagent that was used was of analytical grade.

### 2.2. Extraction and Purification of SFN

The procedure for the extraction and purification of SFN was performed in accordance with the approach that was previously described by Li et al. [29] with minor adjustments. After the freeze-dried broccoli seeds had been pulverized with a pulverizer, the resulting powder was subjected to a treatment at 60 °C for 5 min to deactivate the epithiospecifier protein. The powder was degreased using n-hexane, after which deionized water was added, and the mixture was hydrolyzed in a water bath at 25 °C for 12 h (the ratio of reagent to material was 10 mL/g). Finally, the crude SFN was prepared by extracting with two equal volumes of ethyl acetate, concentrated under vacuum and lyophilized.

The fractions containing SFN were dissolved in deionized water and further purified by semi-preparative high-performance liquid chromatography (HPLC) equipped with an XBridge Prep C18 OBD Prep Column, 19 mm × 150 mm (Waters, Milford, MA, USA), and eluted with an isocratic mixture of methanol and water (at a ratio of 4:6) at a flow rate of 10 mL/min. The temperature of the column oven was set to 30 °C. The wavelength of detection was 201 nm.

### 2.3. Simulated Saliva Digestion

The simulated saliva digestion was evaluated using a modified version of the method previously described [30]. In brief, the simulated saliva was made by dissolving 1.126 g/L of KCl, 1.143 g/L of NaHCO_3_, 0.167 g/L of CaCl_2_, 0.12 g/L of NaCl and 150 U/mL of α-amylase in deionized water, and the pH was adjusted to 7.0 with 0.1 M HCl. There were three sets of samples. Sample A was a mixture of 5.0 mL of simulated saliva and 5 mL of SFN solution (3 mg/mL); sample B was a mixture of 5.0 mL of deionized water and 5 mL of SFN solution (3 mg/mL); sample C was a mixture of 5.0 mL of simulated saliva and 5.0 mL of distilled water. All three sets of samples were placed in a rotary shaker at 37 °C and 110 rpm to mimic saliva digestion. During the digestion process, 2.0 mL aliquots were taken at 0, 2, 4 and 6 h after the digestion, and were immediately heated in a boiling water bath for 10 min to deactivate the enzyme.

### 2.4. Simulated Gastric Digestion

The simulated intestinal digestion was carried out based on a published procedure [31,32] with minor adjustments. The gastric electrolyte solution was prepared by dissolving 310.0 mg NaCl, 110.0 mg KCl, 15.0 mg CaCl_2_ and 60.0 mg NaHCO_3_ in 100 mL deionized water. The pH of the gastric electrolyte solution was adjusted to 2.0 with 0.1 M HCl. The simulated gastric juice was prepared by combining 15.0 mL gastric electrolyte solution, 3.6 mg pepsin, 3.8 mg gastric lipase, and 0.3 mL of 1.0 M CH_3_COONa solution (pH 5.0), with the pH adjusted to 2.0 with 0.1 M HCl. A volume of 15.0 mL of 3.0 mg/mL SFN solution or 15.0 mL of deionized water (blank control) was added to 15.0 mL of simulated gastric juice, and the mixture was incubated in a shaker (100 rpm) at 37 °C for digestion. During the simulated digestion process, 2.0 mL aliquots were taken at 0, 2, 4 and 6 h after the digestion and boiled for 10 min to deactivate the enzyme.

### 2.5. Simulated Small Intestinal Digestion

The simulation of intestinal digestion was carried out with minor modifications to the previously described approach [31,33]. The intestinal electrolyte solution was prepared by dissolving 540.0 mg NaCl, 65.0 mg KCl and 33.0 mg CaCl_2_ in 100 mL deionized water and adjusting the pH to 7.0, using 1 M NaHCO_3_ solution. Then, the simulated small intestinal juice was prepared by mixing 6.5 mg trypsin, 200.0 mL bile salt solution (4%, *w*/*w*) and 100.0 mL of pancreatin solution (7%, *w*/*w*) with 50.0 mL of intestinal electrolyte solution. The pH of the solution was adjusted to 7.5 using 0.1 M NaOH. Lastly, 15.0 mL of gastric digest sample obtained by 6 h digestion was added to 5.0 mL of stimulated small intestinal juice and allowed to react in a thermostatic shaker at 37 °C. During the reaction process, 2.0 mL aliquots were collected at 0, 1, 2, 4 and 6 h for further analysis and heated in a boiling water bath for 10 min to deactivate the enzyme.

### 2.6. In Vitro Fermentation of SFN

In vitro fermentation of SFN from fresh human feces was carried out as previously described [23,34] with minor modifications. On the day of fermentation test, three obese volunteers aged 24–32 years with a BMI of 35 kg/m^2^ provided fresh stool samples. These volunteers had no digestive illnesses and had not taken antibiotics in the three months before the fermentation test; they also voluntarily signed the informed consent form. Our study was performed in accordance with the principles of the Declaration of Helsinki with regard to ethical research involving human subjects, and the protocols were approved by the Medical Ethics Committee of Nanjing Hospital of Integrated Traditional Chinese and Western Medicine (approval No. S2021–10-008). Each volunteer’s three feces samples were blended and diluted with phosphate-buffered saline (0.1 M, pH 7.2) to generate a 10% fecal slurry (*w*/*v*) that was fully homogenized and filtered through four layers of sterile gauze sponge cloth. A mixture of 5 mL filtered fecal slurry and 5 mL phosphate buffer containing 120 mg SFN was injected into the sampling port of the BGR system’s large intestine reactor. The BGR was then flushed with nitrogen three times to remove oxygen before 230 mL BHI medium was added and placed into a sterilization pot at 115 °C for 20 min. Fermentation was conducted in BGR at 37 °C. The samples (10 mL) were collected at 0, 6, 12 and 24 h, and stored in a −80 °C refrigerator until further analysis.

### 2.7. Detection of SFN

Following digestion by saliva or gastrointestinal juice for a defined period of time, the residual SFN was detected using a Waters HPLC system (Waters, Milford, MA, USA), equipped with a ZORBAX SB-Aq C18 column (4.6 mm × 250 mm, 5 μm) (Agilent Technologies, Santa Clara, CA, USA), which was eluted with an isocratic elution consisting of methanol and water at a ratio of 4:6 and a flow rate of 1 mL/min.

The mass spectrum of SFN was detected on an Agilent 1260 Infinity II liquid chromatograph (Agilent Technologies Santa Clara, CA, USA) interfaced with an Agilent 6420 triple quadrupole mass spectrometer (Agilent Technologies Santa Clara, CA, USA). Analysis of SFN in a mass range of 50–200 (*m*/*z*) was performed under the following conditions: negative ion mode; capillary voltage 3.5 kV; nebulizer temperature, 45 psi; gas flow rate, 10 L/min with a temperature of 350 °C [35].

### 2.8. Determination of Optical Density and SCFAs

The optical density (OD) of the collected samples was determined using a Sunrise™ microplate reader (Tecan, Männedorf, Switzerland) at wavelength of 600 nm. The content of SCFAs was determined by the gas chromatography (GC) method as previously described [36] with minor modifications. Briefly, a 1.0 mL sample was mixed with 50 μL of phosphoric acid (34%) to ensure that each organic acid component existed in acid form. SCFA content was analyzed on a Shimadzu GC-2014 system (Shimadzu, Kyoto, Japan) equipped with a flame ionization detector (FID) and DB-FFAP column (30 m × 0.25 mm × 0.25 μm). GC was operated with nitrogen as the carrier gas at a flow rate of 60 mL/min. The temperatures of the injector and the FID were set at 250 °C and 300 °C, respectively. The initial oven temperature was held at 100 °C for 5 min, before being elevated to 250 °C at a rate of 10 °C/min and held for 12 min.

### 2.9. Analysis of Gut Microbiota

The total bacterial DNA of the samples were extracted using TIANamp Stool DNA Kit (Tiangen, Beijing, China) as per the manufacturer’s instruction. The V4 region of bacterial 16S rDNA was sequenced by the Illumina Miseq platform (San Diego, CA, USA). DNA sequence alignment and analysis were undertaken in accordance with the deriving microbial operational taxonomic units (OTUs), from which species richness was estimated as previously described [37].

### 2.10. Statistical Analysis

Each experiment was repeated three times. All data were represented as mean ± standard deviation (SD). One-way analysis of variance (ANOVA) and Tukey’s test (SPSS version 26.0, IBM Inc., Chicago, IL, USA) were used to evaluate the statistical differences, with *p* < 0.05 as the threshold value.

## 3. Results and Discussion

### 3.1. Extraction and Purification of SFN

**Figure 1:**
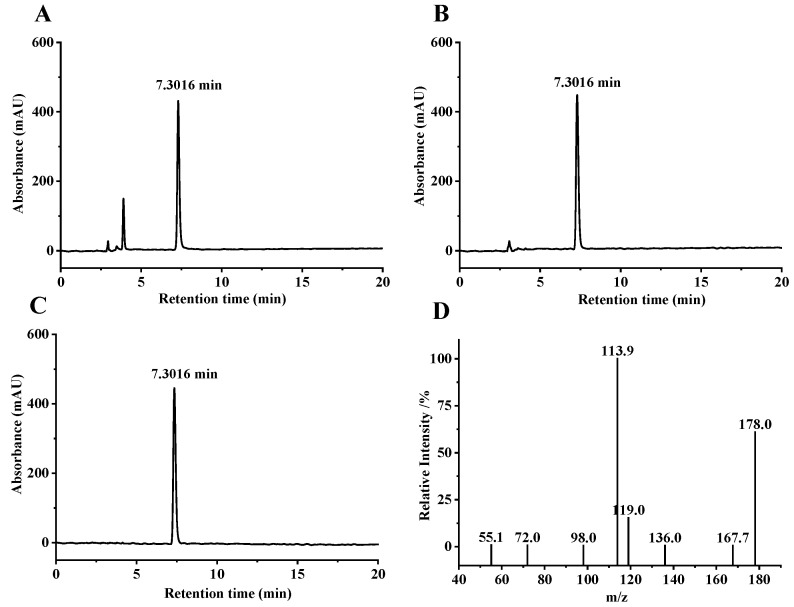
High-performance liquid chromatogram (HPLC) chart of crude sulforaphane (SFN) extract derived from broccoli seeds. (**A**), standard SFN (**B**), purified product (**C**), and secondary mass spectrum (**D**).

From the crude broccoli seed extract and 0.5 mg/mL SFN standard, 200 μL were taken of each, and HPLC was used under the same conditions to obtain liquid chromatograms of SFN crude extract and standard; both displayed a peak at the retention time of 7.3016 min (Figure 1A,B). The peak was consistent in shape without the interference of additional peaks; it was hence conceivably determined that the peak at the retention time of 7.3016 min in the broccoli seed extract represented SFN. SFN was purified by semi-preparative liquid chromatography, and the purified product was determined by HPLC to obtain a liquid chromatogram (Figure 1C). The purity was calculated by using the peak area ratio, and the peak area of the purified product was calculated to account for 95%. Further analysis by ultra-high performance liquid chromatography revealed the secondary mass spectrum of SFN (Figure 1D). The authenticity of the purified product as SFN was further verified by referring to related studies [38] and comparing the molecular formula of SFN C_6_H_11_NOS_2_ and the relative molecular mass of 177.028.

### 3.2. Change of SFN in Digestion of Simulated Saliva

**Figure 2:**
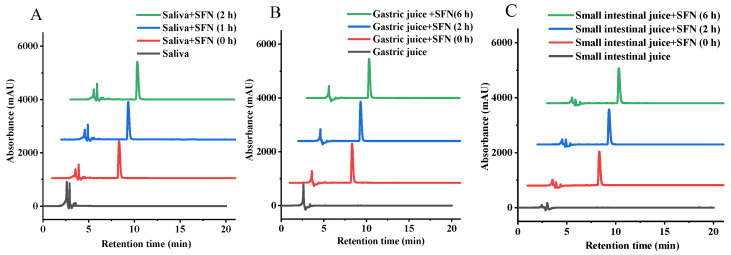
High performance liquid chromatogram (HPLC) of sulforaphane (SFN) after digestion in simulated saliva (**A**), simulated gastric fluid (**B**) and simulated small intestinal fluid (**C**).

The most abundant protein in human saliva is salivary amylase [39], which is the first to contact the sample and degrade the α-(1→4) glycosidic linkages in starch and other carbohydrates. Sulforaphane (SFN) is easily decomposed under alkaline conditions, but relatively stable under acidic conditions. To investigate whether SFN can be digested in saliva, we first assessed the effect of human salivary amylase on SFN. As is evident in Figure 2A and Table 1, neither retention time nor content of SFN changed significantly after saliva digestion, and no new peaks appeared during digestion, indicating that SFN was not degraded during saliva digestion.

### 3.3. Change of SFN in Simulated Gastrointestinal Digestion

In order to answer the question of whether or not SFN could be digested and utilized in the gastrointestinal tract, the digestibility of SFN was evaluated using a fluid model that simulated gastric and small intestinal fluids. As shown in Figure 2B,C, there was no discernible alteration in the retention time and the content of the SFN following the defined period of simulated gastric and small intestinal fluid digestion (Table 1), indicating that the SFN’s resistance to digestion in gastrointestinal intestinal digestion is comparable to that of salivary digestion. It is therefore plausible that SFN can pass through the digestive system without being broken down by either saliva or mimicked digestion in the stomach and small intestine, and that it is delivered to the colon in a relatively unchanged state.

### 3.4. Fermentation In Vitro of SFN by Human Gut Microbiota

**Figure 3:**
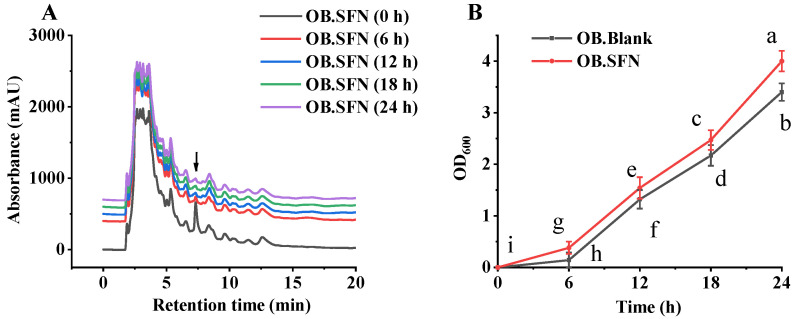
Changes in sulforaphane (SFN) and microflora growth during in vitro fecal fermentation. (**A**) HPLC profiles of SFN during fecal microbiota fermentation in obese patients and (**B**) microflora growth during in vitro fecal fermentation in obese patients. a–i: Mean value in the same group with different letters was significantly different (*p* < 0.05) by a Tukey test. The arrows in (**A**) represent sulforaphane.

The effect of in vitro fermentation of SFN was evaluated by analyzing SFN composition during the fermentation process. As shown in Figure 3A, the content of SFN declined significantly after 6 h of fermentation, and then stabilized after 12 h of fermentation, indicating that SFN can be utilized by gut microbes. As shown in Figure 3B, after 24 h of fermentation, the OD600 of the fecal blank group of obese patients was significantly lower than that of the OB.SF group, which may be related to the fact that SFN promotes the growth of beneficial microorganisms whilst simultaneously inhibiting the growth of pathogenic bacteria [40,41].

### 3.5. Effects of SFN Fermentation In Vitro on SCFAs Production

**Figure 4:**
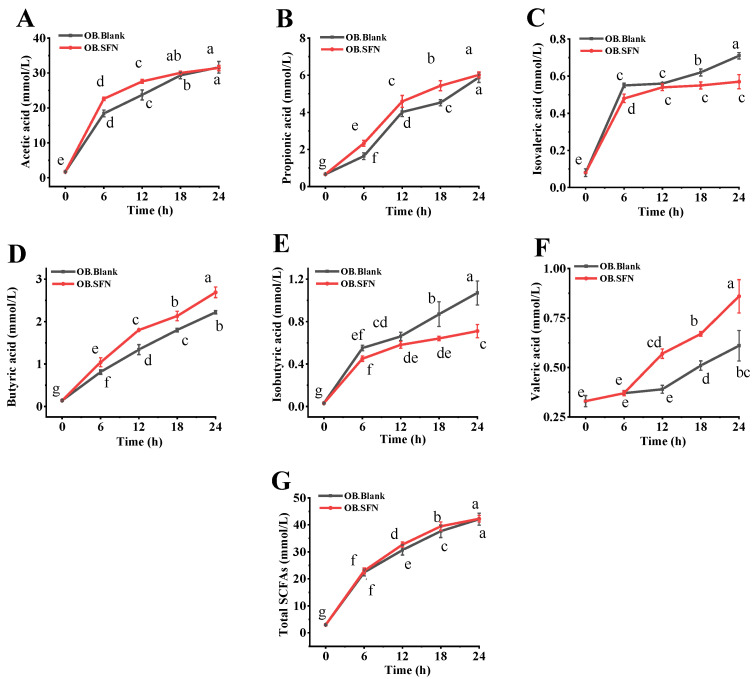
Sulforaphane (SFN) affects the metabolites produced by gut microbiota during fermentation. Concentrations of (**A**) acetic acid, (**B**) propionic acid, (**C**) isobutyric acid, (**D**) butyric acid, (**E**) isovaleric acid, (**F**) valeric acid and (**G**) total short-chain fatty acids (SCFAs). a–g: Different letters indicated significant differences in the same group using Tukey test (*p* < 0.05).

SCFAs, such as acetate, propionate, isobutyrate, butyrate, isovalerate and valerate, are the primary metabolites generated by gut microbial communities. Following absorption in the colon, SCFAs play an important role in maintaining proper intestinal function and the morphology and function of colonic epithelial cells, in addition to storing energy and reducing osmotic pressure [42]. As depicted in Figure 4 and Table S1, after 24 h of fermentation, the concentration of total SCFAs in the OB.SFN group increased from 2.9 ± 0.210 mM at 0 h to 42.29 ± 1.225 Mm, which was slightly higher than that in the OB.Blank group (42.12 ± 2.0370 mM). The OB.SFN group was able to boost the content of propionate, butyrate and valerate by 1.02, 1.21 and 1.46 times, respectively, relative to the control group. Propionate and butyrate are implicated in maintaining colon health [43,44], and propionate can reduce serum cholesterol levels, lipogenesis and cancer development [45]. As the primary source of energy for colon cells, butyrate can prevent bacteria from entering the bloodstream and generating inflammatory responses by fortifying the intestinal mucosal immune barrier. In addition, butyrate can regulate host immunological function and metabolism by exerting anti-inflammatory effects, hence maintaining intestinal homeostasis [46,47]. Valerate is an immunomodulatory and well-tolerated medication with important functions in multiple sclerosis, autoimmune diseases and cancer immunotherapy [48]. In addition, given that bacteria can utilize SFN as a carbon source, the absence of an external carbon supply in the OB.Blank group may have contributed to its significantly greater amounts of isobutyrate and isovalerate compared to the OB.SFN group (Figure 4C,E). Both isobutyrate and isovalerate are recognized to be detrimental to colon and metabolic health. Therefore, the ingestion of SFN has led to improvements in the gut microbiome and the gut microbial environment.

### 3.6. Effect of SFN on Gut Microbiota

**Figure 5:**
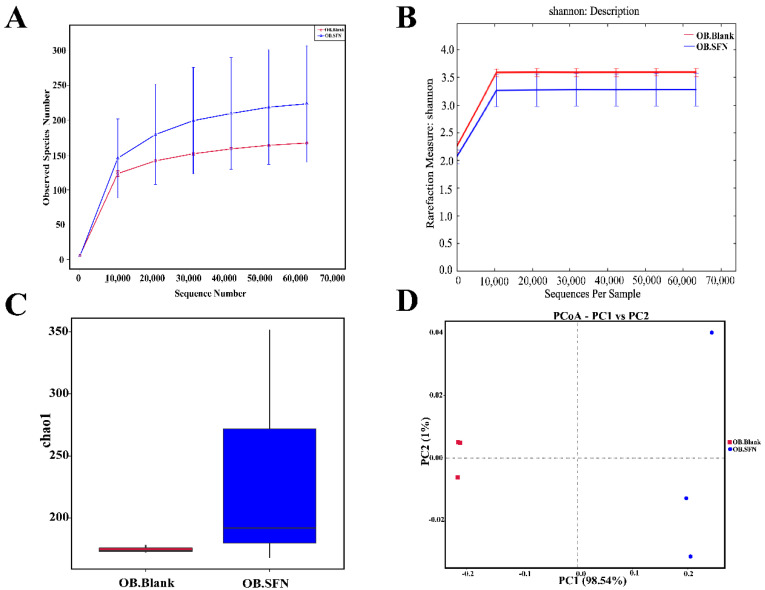
The effect of sulforaphane (SFN) on the microbial community of obese patients at 24 h of fermentation. (**A**) Rarefaction curve of the sample; (**B**) Shannon curve of the sample; (**C**) Chao1: total number of bacterial species; (**D**) principal component analysis (PCA) plot.

To explore the effect of SFN on the makeup of the gut microbiota, 16S rDNA was isolated and analyzed 24 h after fermentation. A total of 421,566 valid reads were obtained from the two groups (the OB.SFN group and the OB.Blank group, each with three replicates), with an average of 70,261 reads per sample. As shown in Figure 5A,B, the sparsity and Shannon index curves gradually flattened, indicating that the amount of data was sufficient to reflect most of the biological information in each sample, hence validating the accuracy and reliability of the analyzed data. Further, as shown in Figure 5C, the expression of CHAO1 that is a marker of bacterial community richness was substantially higher in the OB.SFN group than in the OB.Blank group, suggestive of the SFN’s involvement in promoting the proliferation of gut microbes. PCA revealed distinct gut microbial community composition among groups (Figure 5D). According to the relative abundance of OTUs, the two main axes revealed a difference of 99.54% (PC1: 98.54% and PC2: 1%) between OB.Blank and OB.SFN, demonstrating that SFN significantly influences the organization of the gut microbial community.

**Figure 6:**
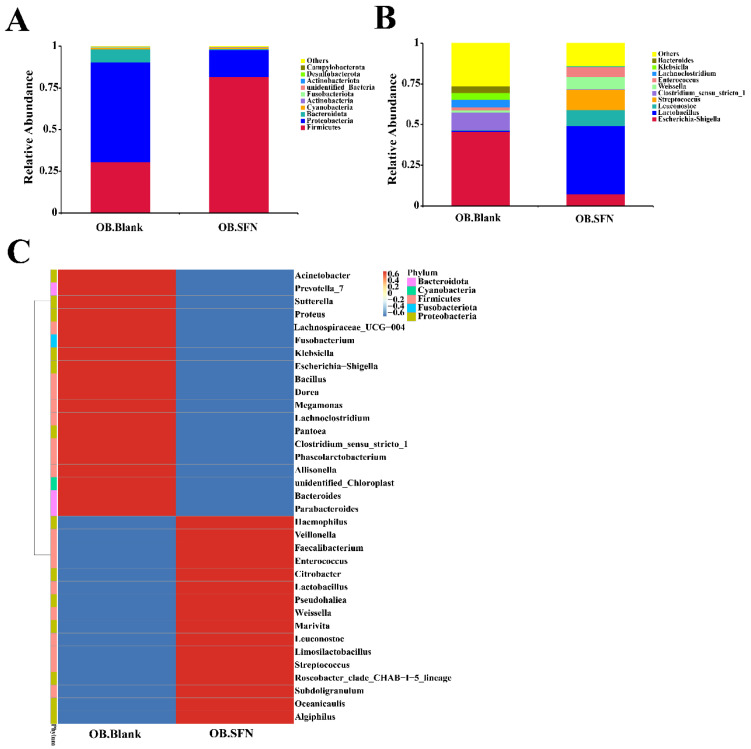
The effect of sulforaphane (SFN) on the gut microbiota in obese patients. (**A**) Gut microbial composition at phylum-level; (**B**) gut microbial composition at genus-level; (**C**) heatmap of gut microbial composition at genus-level.

The phylum-level alterations in gut microbes in obese patients are shown in Figure 6A, which reveals that *Bacteroidetes*, *Firmicutes* and *Proteobacteria* were the predominant flora. After 24 h in vitro fermentation, the SFN-treated experimental group had significantly more abundant *Firmicutes* and significantly fewer *Proteobacteria* compared to the OB.Blank group. It has been established that *Firmicutes* are the predominant beneficial bacteria in the human gut microflora [23]. *Proteobacteria* is a classic indicator of intestinal flora imbalance, resulting in a pathogenic response in the human body [49]. Therefore, the reduction in its abundance from 60.287% to 16.252% in the OB.SFN group appears to be a favorable development as the result of SFN ingestion.

We further analyzed the relative proportion of bacteria at the genus level. As shown in Figure 6B, the relative abundances of *Lactobacillus*, *Leuconostoc* and *Weissella* increased substantially. Among them, *Lactobacillus* is well known for its role in maintaining and regulating the flora of the gastrointestinal tract, leading to desirable immunity enhancement, protection of gastric mucosa, improvements in digestion, and suppression of tumor growth [50]. *Leuconostoc* has antagonistic effects against common pathogens such as *Shigella*, *Salmonella* and *Staphylococcus aureus* [51]. In addition, the relative abundances of *Escherichia-Shigella*, *Klebsiella* and *Clostridium_sensu_stricto_1*, most of which are known to be pathogenic, decreased significantly [52]. These findings imply that SFN promotes the proliferation of beneficial bacteria and inhibits the growth of harmful bacteria.

The relative abundance of the top 35 genera is shown in Figure 6C. To determine the variations in community composition between the OB.Blank and OB.SFN groups, genera with relative abundances below 1% were omitted from the analysis. In comparison to the control group, SFN treatment bolstered the relative abundances of *Weissella*, *Leuconostoc*, *Lactobacillus*, *Algiphilus* and *Faecalibacterium*, and reduced the relative abundances of *Escherichia-Shigella*, *Klebsiella*, *Clostridium_sensu_stricto_1*, *Sutterella*, *Megamonas* and *Proteus*. It has been reported that *Algiphilus* can produce succinic acid and a small amount of acetic and propionic acids, and that succinic acid can activate intestinal gluconeogenesis, imparting beneficial effects on blood sugar metabolism and obesity management [53]. The genus *Faecalibacterium* is one of the most important bacteria in the human gut flora and one of the important producers of butyric acid, which has anti-inflammatory and protective effects on the digestive system from intestinal pathogens, particularly effective in obese patients [54,55].

As the core bacteria in the gut, *Megamonas* are intimately associated with inflammatory bowel disease, colorectal cancer, autism spectrum disorder (ASD), obesity and a number of other conditions. Compared to healthy individuals, the levels of *Megamonas* in the guts of obese and ASD patients are significantly higher [56,57]. *Sutterella* is one of the most prevalent *Proteobacteria*, and its relative abundance was dramatically elevated in obese patients, implying its involvement in the onset and development of obesity [58,59]. In general, we have demonstrated that SFN can improve the composition of human gut microflora by suppressing the growth of pathogenic bacteria, while maintaining the equilibrium of beneficial intestinal flora.

## 4. Conclusions

In this study, crude SFN was extracted from broccoli seeds and refined utilizing a semi-preparative liquid phase to get SFN that was 95% pure. SFN survived simulation of saliva and gastrointestinal digestion, showing that it can reach the colon intact undamaged. In vitro bionic intestinal reactor fermentation results showed that SFN boosted butyric acid and valeric acid increased 1.21-fold and 1.46-fold, respectively. It was further demonstrated that SFN can be utilized by gut microbes to promote the formation of short-chain fatty acids via in vitro fermentation. Furthermore, SFN was shown to increase the abundance of numerous beneficial intestinal bacteria, including *Weissella*, *Leuconostoc*, *Lactobacillus*, *Algiphilus* and *Faecalibacterium*, whist inhibiting the proliferation of harmful bacteria, such as *Escherichia-Shigella*, *Klebsiella*, *Clostridium_sensu_stricto_1*, *Sutterella*, *Megamonas* and *Proteus*. These findings not only shed additional light on the mechanisms that underpin the health benefits of SFN, but also provide a potentially feasible revenue for ameliorating the symptoms of obesity by modifying the makeup of the gut microbiota.

## Figures and Tables

**Table 1:** Contents of SFN at different time points following saliva, simulated gastric and small intestinal digestion.

Process	Digestion Time (h)	Content of SFN (mg/mL)
Saliva digestion	0	1.5139 ± 0.0037 ^a^
	1	1.5165 ± 0.0035 ^a^
	2	1.5133 ± 0.0028 ^a^
Gastric digestion	0	1.6136 ± 0.0059 ^a^
	2	1.6140 ± 0.0027 ^a^
	6	1.6133 ± 0.0020 ^a^
Small intestinal digestion	0	1.2780 ± 0.0024 ^a^
	2	1.2768 ± 0.0015 ^a^
	6	1.2751 ± 0.0030 ^a^

^a^ Mean value in the same column with different letters was significantly different (*p* < 0.05) by Tukey’s test.

## Data Availability

Data is contained within the article.

## Supplementary Materials

The following supporting information can be downloaded at: https://www.mdpi.com/article/10.3390/foods11244016/s1, Table S1. The concentrations of SCFAs in fermentation solutions at different time points of fermentation.
