# Supplementary material for: Simulated Digestion and Fermentation In Vitro by Obese Human Gut Microbiota of Sulforaphane from Broccoli Seeds
Source: Foods. 2022 Dec 12;11(24):4016. doi: 10.3390/foods11244016 (PMC9778330; doi:10.3390/foods11244016)
Supplement: Supplementary file 1 [file foods-11-04016-s001.zip › foods-2060660-supplementary.pdf]

Table S1 The concentrations of SCFAs in fermentation solutions at different time points of fermentation

Sample	Time (h)	SCFAs (mM)						
		Acetic acid	Propionic acid	isobutyric acid	butyric acid	isovaleric acid	valeric acid	Total
OB.Blank	0	1.73±0.096[e]	0.66±0.011[g]	0.08±0.021[e]	0.14±0.018[g]	0.03±0.004[g]	0.33±0.028[e]	2.96±0.178[g]
	6	18.42±0.970[d]	1.64±0.179[f]	0.55±0.012[c]	0.81±0.054[f]	0.55±0.026[ef]	0.37±0.013[e]	22.35±1.255[f]
	12	23.73±1.417[c]	4.02±0.253[d]	0.56±0.007[c]	1.34±0.118[d]	0.66±0.039[cd]	0.39±0.020[cd]	30.69±1.854[e]
	18	29.33±0.952[b]	4.52±0.165[c]	0.62±0.019[b]	1.8±0.043[c]	0.87±0.116[b]	0.51±0.024[d]	37.65±2.315[c]
	24	31.53±1.521[a]	5.88±0.271[a]	0.71±0.017[a]	2.22±0.040[b]	1.07±0.113[a]	0.61±0.077[a]	42.02±2.037[a]
OB.SFN	0	1.70±0.110[e]	0.67±0.021[g]	0.07±0.022[e]	0.14±0.020[g]	0.03±0.005[g]	0.34±0.028[e]	2.95±0.210[g]
	6	22.62±0.498[d]	2.33±0.158[e]	0.48±0.023[d]	1.04±0.106[e]	0.45±0.025[f]	0.37±0.012[e]	27.29±0.390[f]
	12	27.62±0.571[c]	3.59±0.320[c]	0.54±0.018[c]	1.80±0.011[c]	0.58±0.032[de]	0.57±0.024[e]	34.71±0.977[d]
	18	30.06±0.752a[b]	5.43±0.270[b]	0.55±0.019[c]	2.13±0.112[b]	0.64±0.022[de]	0.67±0.012[b]	39.48±1.619[b]
	24	31.43±1.339[a]	6.02±0.164[a]	0.57±0.038[c]	2.69±0.125[a]	0.71±0.062[c]	0.86±0.084[bc]	42.29±1.225[a]

[a~g] Mean value in the same column with different letters shows significant differences by a Tukey test ($P < 0.05$).
